# Basic Instinct Undressed: Early Spatiotemporal Processing for Primary Sexual Characteristics

**DOI:** 10.1371/journal.pone.0069726

**Published:** 2013-07-19

**Authors:** Lore B. Legrand, Marzia Del Zotto, Rémi Tyrand, Alan J. Pegna

**Affiliations:** 1 Laboratory of Experimental Neuropsychology, Neuropsychology Unit, Neurology Clinic, Geneva University Hospitals, Geneva, Switzerland; 2 Faculty of Psychology and Educational Science, University of Geneva, Geneva, Switzerland; 3 Neurology Clinic, Geneva University Hospitals, Geneva, Switzerland; Cuban Neuroscience Center, Cuba

## Abstract

This study investigates the spatiotemporal dynamics associated with conscious and non-conscious processing of naked and dressed human bodies. To this effect, stimuli of naked men and women with visible primary sexual characteristics, as well as dressed bodies, were presented to 20 heterosexual male and female participants while acquiring high resolution EEG data. The stimuli were either consciously detectable (supraliminal presentations) or were rendered non-conscious through backward masking (subliminal presentations). The N1 event-related potential component was significantly enhanced in participants when they viewed naked compared to dressed bodies under supraliminal viewing conditions. More importantly, naked bodies of the opposite sex produced a significantly greater N1 component compared to dressed bodies during subliminal presentations, when participants were not aware of the stimulus presented. A source localization algorithm computed on the N1 showed that the response for naked bodies in the supraliminal viewing condition was stronger in body processing areas, primary visual areas and additional structures related to emotion processing. By contrast, in the subliminal viewing condition, only visual and body processing areas were found to be activated. These results suggest that naked bodies and primary sexual characteristics are processed early in time (i.e., <200 ms) and activate key brain structures even when they are not consciously detected. It appears that, similarly to what has been reported for emotional faces, sexual features benefit from automatic and rapid processing, most likely due to their high relevance for the individual and their importance for the species in terms of reproductive success.

## Introduction

The necessity to reproduce is a basic process whose existence ensures the reproductive success of the species [1]. The drive for sexual satisfaction, and thus the urge to mate, is on par with the need for nutritional supply, fluid balance, maintenance of energy, thermoregulation and defense [1], [2]. Within this context, a motivational state is normally present in the organism (sexual desire, hunger etc.), which provides the incentive that can lead to consummation (sexual activity, eating) [2], [3]. Consequently, our nervous system is likely genetically equipped with the basic processing circuits allowing us to detect relevant sexual stimuli and ensuring that we are rapidly and automatically tuned to those that are behaviorally relevant to our basic needs. We could therefore expect visual sexual stimuli (VSS) to belong to a category of stimuli that might produce patterns of activation different from less meaningful stimuli, perhaps benefitting from enhanced processing as has been described for emotional stimuli [4], [5], [6], [7].

Visual stimuli are initially processed by the primary visual cortex, located in the occipital lobe and subsequently through two separate pathways located in the temporal and the parietal cortex, which deal respectively with visual and spatial features [8]. Subsequently, body stimuli were found to be processed in category-specific occipito-temporal areas, which include the extrastriate body area (EBA) and the fusiform body area (FBA) [9], [10], [11], [12], [13], [14]. Most noteworthy however, studies that have compared sexual and non-sexual visual stimuli have found that the former produce a stronger response in primary sensory regions, as well as the EBA and FBA. These last two structures were found to increase their activity in response to naked bodies, emotionally expressive bodies, and erotica (photos or film excerpts) [15], [16], [17], [18].

Sexual stimuli are also known to activate a network that extends beyond strictly “visual” areas, encompassing what has been termed a “sexual interest network” [2]. Indeed, the initial assessment of a desirable stimulus in the sexual response cycle [19] appears to include the ventral striatum/nucleus accumbens and amygdala, thought to shape the motivational aspect. In addition, the hypothalamus, orbitofrontal cortex (OFC), pregenual anterior cingulate cortex and anterior insula have also been found to be implicated for more elaborate, subjective processes as for example attractiveness judgments [20] or the coupling of externally perceived emotional states and internal emotional states [21].

One aspect that has received less attention is the particular status of VSS as an evolutionary relevant category of stimuli entailing the possibility that it may be processed rapidly and without awareness through a subcortical pathway. Indeed, it has been suggested that an innate and hard-wired subcortical pathway might be automatically activated to perform an initial fast and crude appraisal of behaviorally relevant stimuli [1], [22], such as for example emotional faces [23]. In the visual modality, this rapid route would run in parallel to the main, geniculo-striate processing route, bypassing the primary sensory areas and projecting directly to the amygdala via the superior colliculus and pulvinar. The existence of this pathway has been suggested and investigated in detail for emotional faces in human subjects [4], [6], [7], [24]. However, behavioral relevance is certainly not restricted to emotional faces and sexual stimuli could constitute perfect targets for such a pathway. Indeed, one could hypothesize that the amygdala possesses a rough representation of salient sexual body characteristics that could be accessed non-consciously through the same subcortical pathway.

In fact, a few lines of evidence do suggest that non-conscious processing could occur for sexual stimuli. In behavioral paradigms, subliminal sexual primes were shown to facilitate the categorization of subsequent sexual targets. Sexual stimuli were categorized as such more rapidly when preceded by a sexual prime than a neutral one, even when the latter was presented subliminally [25]. In another report using an attention-shifting paradigm, naked bodies were rendered non-conscious through binocular rivalry. Despite the lack of awareness of the stimuli, photographs of naked opposite-sex bodies captured the attention of heterosexual male and female participants, while the converse effect (attraction of attention by same-sex bodies) was observed in homosexual viewers [26].

Using fMRI, supraliminal and subliminal presentations of sexual stimuli (pictures of nudes with faces compared to abstract drawings) showed differential patterns of activation. Subliminal stimuli essentially produced greater activation in areas implicated in sexual arousal, such as the caudate nucleus and thalamus, while supraliminal presentations produced additional activity notably in frontal areas associated with behavioral control [27]. These results were taken to show that subliminal presentations circumvent top down inhibitory processes that might occur when sexual stimuli are presented.

These findings show that naked bodies can be processed subliminally and activate specific brain areas. However none of these paradigms has specifically addressed the temporal aspect of activation in an attempt to establish the existence of a rapid subcortical processing for this type of stimuli. In addition, most studies investigating VSS have varied several dimensions of the images simultaneously, often presenting individuals during sexual interactions in which the stimuli could include moving stimuli (or static but suggesting implicit movement), different emotional expressions and states, or more or less sexually expressive postures [28], [29], [30]. Importantly, in some of these studies the primary sexual characteristics were not clearly visible.

Only one very recent study has investigated the effect of solitary static naked bodies devoid of any marked sexually suggestive expression in EEG under supraliminal viewing conditions. In this investigation, the presentation of naked bodies in neutral positions was found to enhance an early component, the N1, when compared to dressed bodies [31].

In view of the behavioral importance of sexual features and the possibility that, as for emotional faces, they might be processed very rapidly and without awareness, we decided to investigate the ERP response for naked bodies. In particular we meant to replicate the only existing study showing an N1 response for naked bodies [31], and more importantly to establish whether this response also holds under subliminal viewing conditions. We hypothesized firstly that in the conscious (supraliminal) viewing condition, sexual characteristics of the preferred sex would enhance the very early N1 component due to possible rapid access to the amygdala through the subcortical pathway, and more importantly that the same effect would arise even in the unconscious (subliminal) viewing condition. We therefore presented supraliminal and subliminal backward masked stimuli to heterosexual participants representing either naked or dressed men or women in neutral positions. High-density electroencephalography (EEG) was measured during this procedure in order to track fast neuronal processes related to the automatic processing of such stimuli. Naked bodies elicited a more enhanced N1 component than dressed bodies in the supraliminal condition. Most of all, this effect was also found in subliminal viewing conditions for opposite-sex stimuli.

## Methods

### Materials and Methods

#### 2.1. Subjects

Twenty-two healthy subjects (11 females) volunteered to participate in the experiment. Participants were considered heterosexual based on their responses on a 7 point Kinsey scale questionnaire [32]. Two subjects (one male) were subsequently excluded from the study due to the presence of a migraine in one case and numerous blink artifacts in the other. Average age was 26 years (standard deviation 3.7). The study was approved by the Ethics Committee of Geneva University Hospitals and participants gave their written informed consent to participate before beginning the experiment. Participants had normal or corrected-to-normal vision and had no history of neurological or psychiatric disorder. The 20 remaining subjects were right-handed, except for one ambidextrous person (median of the laterality index: 94.7; range: 35–100). Half of the female subjects were using oral contraceptive medication. The sample of subjects did not allow for classification into different groups as a function of menstrual cycle due to the heterogeneous distribution and an insufficient sample size.

#### 2.2. Stimuli

Forty grayscale pictures of dressed and naked bodies posing in a neutral position with their arms hanging down on either side of the body were designed using the character modeling program N-sided Quidam3 (www.n-sided.com) that is dedicated to professional game developers. Pairs of the same “person” were chosen once naked and once dressed. All bodies were of the same height and were all placed on the same medium grey background. Skin color was varied from light (Caucasian) to dark (African) in parallel with the variations in light intensity produced by clothing. Females were designed to show four different hip to waist ratios (1, 1.2, 1.4, 1.6) and men four waist to shoulder ratios (0.75, 0.7, 0.65, 0.6). Faces were manually blurred using the Adobe Photoshop CS3 smudge tool to assure explicit body processing independently of facial feature or expression processing. Scrambled bodies were created by randomly scrambling squares of 20×20 pixels in eight dressed bodies (4 females, 4 males) which were then used as masks.

All stimulus categories were analyzed with the program ImageJ (http://rsbweb.nih.gov/ij/index.html) and adjusted in Adobe Photoshop CS3 to maintain similar luminance across categories. Stimuli (282×785 pixels) were presented in the centre of the CRT monitor (21″ Hewlett Packard) at a refresh rate of 60 Hz, situated at 115 cm from the subject in a Faraday cage with minimal lighting and noise and subtended a visual angle of 5.4° on the horizontal axis by 17.4° on the vertical axis. The experimental procedure was controlled and reaction times were recorded with the psychology software tool E-prime (v.1.2; www.pstnet.com/eprime).

#### 2.3. Procedure

**Figure 1:**
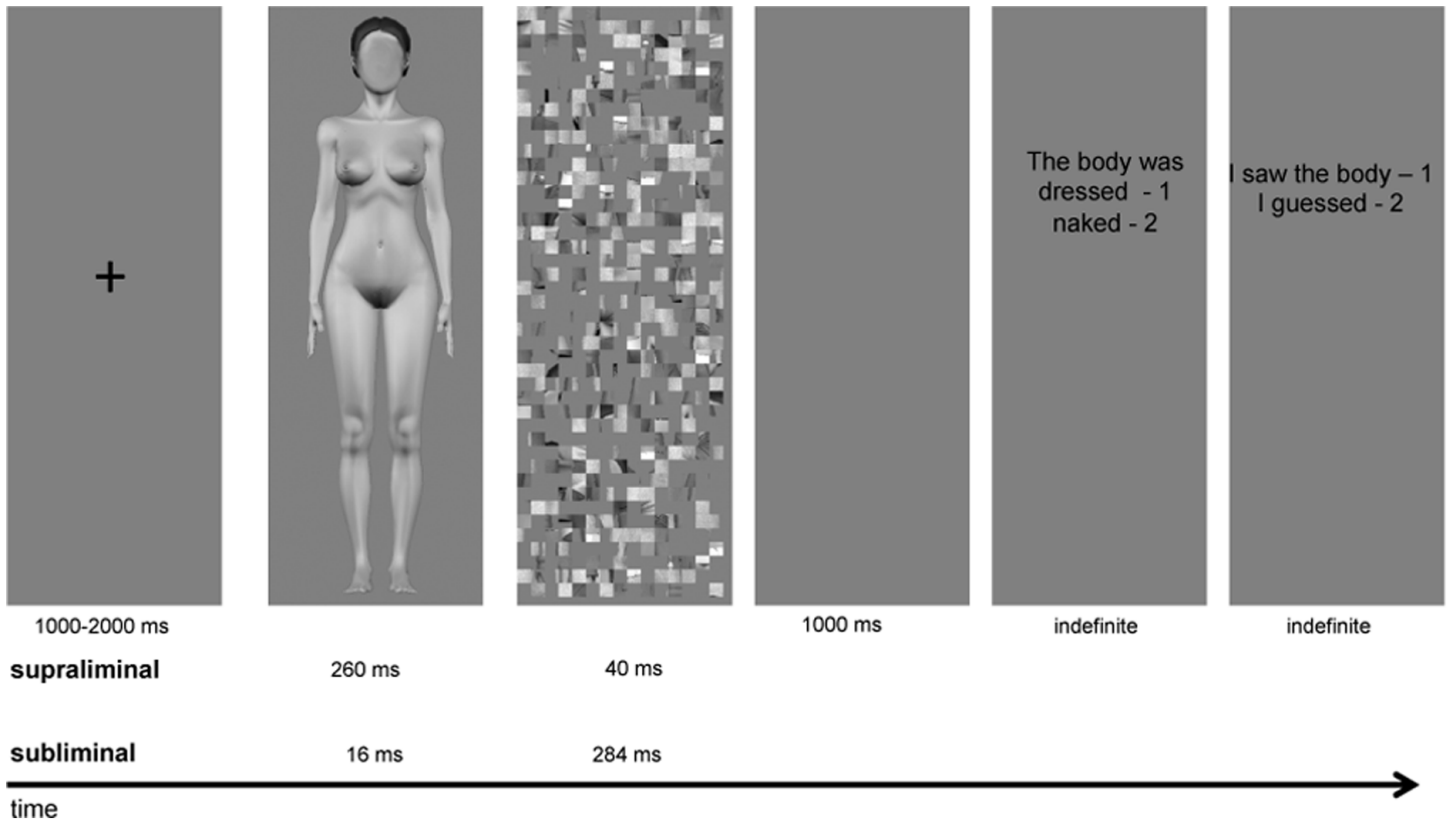
Experimental procedure. An initial fixation cross was presented for random durations between 1000 to 2000 ms, followed by a target for 260 ms in the supraliminal condition and 16 ms in the subliminal condition, followed by a mask for respectively 40 or 284 ms. The questions appeared after a 1000 ms delay. The participants answered by pressing a key, which then prompted the second question and subsequently initiated the next trial.

Pictures of dressed and naked bodies were presented supraliminally and subliminally to participants who were asked to give a response on each occasion. The procedure is detailed in Fig. 1. Every trial began with the presentation of a fixation cross that was randomly presented for durations between 1000 ms to 2000 ms. Next, the target stimulus of a naked or dressed body was presented randomly either for 240 ms (supraliminal) or 16 ms (subliminal) condition. Targets were immediately followed by a mask, composed of a scrambled dressed body whose duration was adjusted so that the target+mask duration was always of 300 ms. Thus, in the subliminal condition, the mask lasted 284 ms and supraliminal condition, the mask lasted 40 ms.

A 1000 ms grey screen was then presented followed by 2 response prompts. This was done to allow for stimulus processing without contamination of the electrical signal by the motor responses. The first prompt asked if the body was dressed or naked. If the subject could not tell, s/he was asked to guess. Participants answered by pressing one of two keys on a keyboard with their middle or index finger. This produced the second prompt asking whether they had based their previous answer on a guess or if they were certain. There was no time constraint and reaction time was therefore not analyzed.

The paradigm was a 2×2×2 design with the following factors: nudity (dressed vs. naked); sex of the stimuli (opposite-sex stimuli –OSS– vs. same-sex stimuli –SSS–)and type of presentation (supraliminal vs. subliminal). The opposite sex stimuli (OSS) condition included the ERPs of female participants to the presentation of male models and of male viewers to female models. The converse constituted the same sex stimuli (SSS) condition. A total of 800 stimuli were presented randomly (10 trials×40 stimuli×2 durations), with a break after every block (1 trial×40 stimuli×2 durations). One block lasted about 5 minutes.

Before the EEG recording began the participants filled out four questionnaires. One assessing the laterality index [33], one assessing their sexual orientation [32], an in-house EEG questionnaire assessing the presence of neurological or psychiatric diseases, the period of menstruation for women and the use of contraceptive methods. Finally, a questionnaire assessing their use of pornographic material was administered. Participants were informed that the study investigated the neural basis of human body processing using dressed and naked male and female bodies. Instructions were to try to detect the body appearing after the fixation cross and determine whether it was dressed or naked. A familiarization run was carried out prior to the experiment proper.

#### 2.4. EEG–ERP recording and analysis

Continuous EEG recorded from 128 scalp electrodes distributed evenly across the scalp surface (referenced to the vertex) was acquired at 2048 Hz using the Biosemi recording software (BioSemi B.V., Amsterdam, Netherlands). Eye movements were monitored using 3 EOG leads. Impedances were kept below 50 kΩ. Using the EEG processing software Cartool (brainmapping.unige.ch/cartool), the EEG was downsampled to 1024 Hz, filtered offline from 0.09 to 50 Hz and recalculated against the average reference [34]. ERP epochs were computed from 100 ms prestimulus to 1000 ms post-stimulus onset for the eight conditions: dressed – naked stimuli; opposite-sex stimuli – same-sex stimuli; supraliminal – subliminal presentation mode. EEG traces with amplitudes exceeding ±100 µV were automatically excluded while other artefacts (including eye blinks) were rejected based on visual inspection.

**Figure 2:**
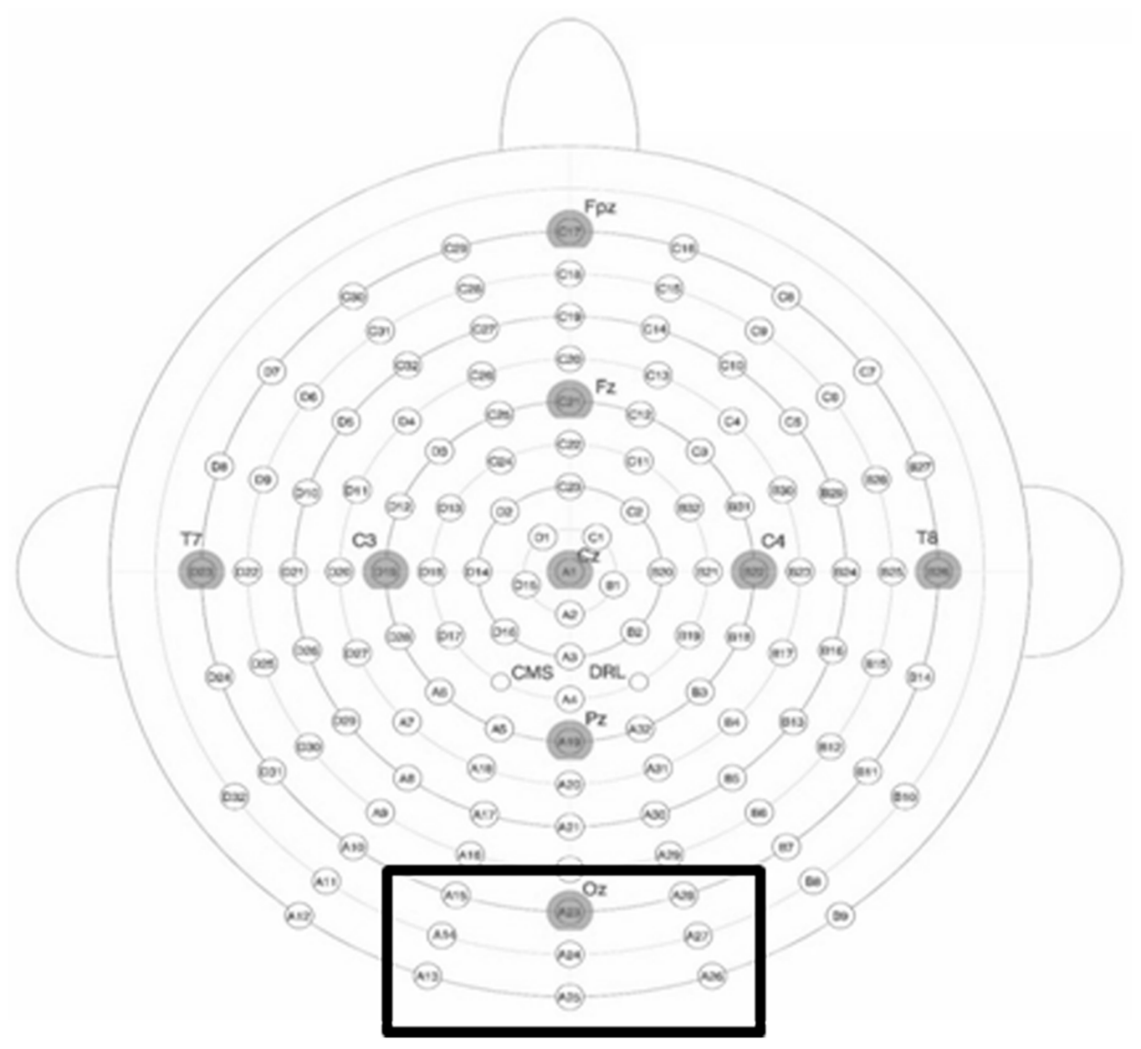
EEG 128 electrode placement (Biosemi). The black square delineates the electrodes of interest.

The electrodes of the region of interest responding to category specific modulation of the electrophysiological response were defined visually on the basis of the grand-mean. A cluster of 9 occipito-temporal electrodes that have also previously been shown to be involved in emotion and body processing [35], [36], were chosen and averaged together. The corresponding electrodes are outlined in Fig. 2. Because of the focus on early component modulation in this study, peak analysis on the N1 component in these regions of interest was run using Brain Vision Analyser’s semi-automatic local maximum detection (Brain Products) using a 10 ms time window centered at the peak of the grand average. Peak amplitudes and latencies in the different categories were tested statistically using a standard analysis of variance (ANOVA) for repeated measures. Data were entered into a 2×2×2 ANOVA which included presentation mode (supraliminal vs. subliminal), nudity (naked vs. dressed bodies) and stimulus-sex (OSS vs. SSS) as repeated measures. One outlier was excluded from the analysis due to amplitudes that differed by over 1.5 standard deviations from the group mean, resulting in N = 19 (10 females). In addition, t-tests were employed to investigate contrasts related to the main hypothesis of this study (effect of nudity independently for each mode of presentation).

#### 2.5. Source localization analysis

The neuronal generators of the N1 peak were estimated using the smooth prior inverse solution implemented in the statistical parametric mapping (SPM8) software (Wellcome Department of Imaging Science; http://www.fil.ion.ucl.ac.uk/spm) that is similar to LORETA (low resolution brain electromagnetic topography) and described in detail elsewhere [37]. This source localization algorithm uses a 3-dimensional grid head model with electric sources distributed on each grid point, computing the smoothest of possible solutions to take into account neuronal synchronicity through co-activation of neighboring grid points. Uncorrected pairwise t-tests were used to compare the inverse solutions voxel by voxel statistically across conditions.

Source estimations of the intracranial generators were computed within a 10 ms time window centered on the peak of the grand average N1 for each of the 8 conditions (dressed/naked; OSS/SSS; supraliminal/subliminal) for every subject separately. Dressed and naked stimuli were then compared statistically by means of a paired t-test, thresholding the clusters at >15 voxels. Separate t-tests were run for the 4 comparisons (i.e., for the same-sex stimuli/opposite-sex stimuli in supraliminal/subliminal conditions).

## Results

### Behavior

The hit rate for naked vs. dressed bodies was at 99% in the supraliminal condition and at 58% in the subliminal condition. Behavioral performance was further analyzed using d’ statistics. The corresponding d’ values are 3.17 in the supraliminal condition and 0.29 in the subliminal condition. A binomial test was performed to compare whether the number of hits in the subliminal condition was significantly different from chance (50%). Responses did not differ significantly from randomness (p>.9). Thus, bodies were clearly identified in the supraliminal condition while correct responses were at chance level in the subliminal condition. This was in line with the answers given for the second question, in which subjects generally claimed that they saw the bodies in almost all (98%) supraliminal trials but guessed their answer in most (88%) trials at 16 ms.

### ERP N1 Analysis

Latency and amplitude values for the N1 in each condition are given in Table 1.

**Table 1:** Result for N1 component.

		latency:		amplitude:	
OPPOSITE SEX					
presentation	Stimuli	mean ms	sE	mean µV	sE
supraliminal	dressed	148	1.3	−4.35	0.9
	naked	148	1.2	−6.11	0.9
subliminal	dressed	147	1.3	−7.70	1.3
	naked	146	1.3	−8.39	1.3
**SAME SEX**					
**presentation**	**Stimuli**	**mean ms**	**sE**	**mean µV**	**sE**
supraliminal	dressed	148	1.1	−4.71	0.9
	naked	149	1.1	−5.85	0.9
subliminal	dressed	148	1.4	−8.01	1.2
	naked	147	1.3	−8.07	1.2
	average	148			

N1 component results: Descriptive statistics for latency and amplitude peak values for OSS and SSS in the supraliminal and subliminal condition.

#### Latency

No significant differences in N1 peak latencies were found in the 2 (presentation mode: supraliminal vs. subliminal) X 2 (nudity: naked vs. dressed) X 2 (stimulus-sex: OSS vs. SSS) ANOVA. The main effect of presentation mode was marginally significant (F(1, 18) = 4.26, p = .054) while nudity (F(1, 18) = .54, p>.1) and stimulus-sex (F(1, 18) = 3.12, p>.05) were not. In addition, neither the first order, nor the second order interactions were significant (all p>.1). Mean latency for the N1 peak was at 148 ms.

#### Amplitude

The 2 (presentation mode) X 2 (nudity) X 2 (stimulus-sex) ANOVA showed significant main effects of presentation mode (F(1, 18) = 14.3, p = .001) and nudity (F(1, 18) = 19.41, p<.001) but not stimulus-sex (F(1, 18) = .12, p>.1). This was due the fact that the N1 component was globally more enhanced for subliminal than supraliminal presentations, as well as for naked compared to dressed bodies.

The interaction between presentation mode and nudity was significant (F(1, 18) = 21.98, p<.001) due to the fact that the difference in amplitude between naked and dressed bodies was greater in the supraliminal condition (dressed supraliminal *M* = −4.56 µV, naked supraliminal *M* = 5.98 µV) than in the subliminal condition (dressed subliminal *M* = −7.85 µV, naked subliminal *M* = −8.23 µV). Post-hoc LSD tests showed that both in the supraliminal and subliminal conditions, dressed bodies differed significantly from naked bodies (both p<.05). In addition, the interaction between stimulus-sex and nudity was significant (F(1, 18) = 10.15, p = .005) due to a greater difference in N1 amplitude between dressed and naked opposite-sex bodies (OSS dressed *M* = −6.02 µV, OSS naked: *M* = −7.25 µV) than dressed vs. naked same-sex bodies (SSS dressed *M* = −6.39 µV, SSS naked: *M* = −6.96 µV). Post-hoc LSD tests showed that both for OSS and SSS, N1 amplitudes differed significantly between dressed and naked conditions (p = <.001). Opposite- vs. same-sex naked bodies only showed a marginal effect (p = .06).

Finally, the three way interaction was not significant (F(1, 18) = .02, p>.1).

Thus our data show that the N1 is enhanced globally for subliminal presentations (main effect) and for naked bodies (main effect). Furthermore, the difference in amplitude between dressed and naked bodies is greater in the supraliminal condition than in the subliminal condition (nudity X presentation mode). Additionally, the increase in N1 observed for naked bodies (compared to dressed bodies) was greater in the OSS than in the SSS (nudity X stimulus-sex). It therefore appears that naked bodies increase the N1 amplitude, and more markedly for opposite-sex bodies.

In order to establish whether this enhancement for naked bodies of the opposite sex appear equally in the supraliminal and subliminal conditions, we performed separate pairwise comparisons on the N1 peak values for each of these presentation modes using standard t-test analyses.

#### Amplitude in supraliminal condition

**Figure 3:**
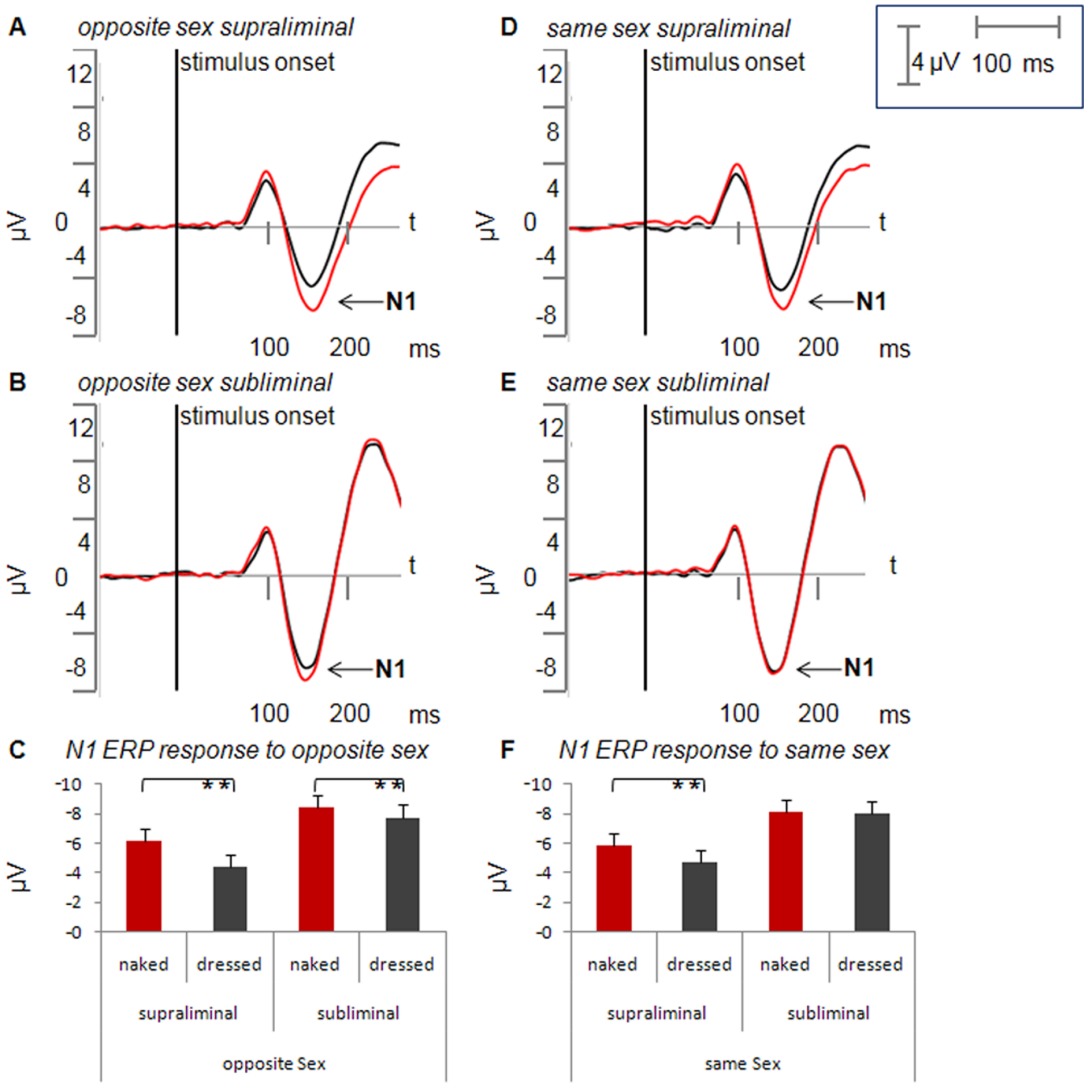
Average ERP for dressed and naked bodies in the supraliminal and subliminal conditions. Average ERP (in µV) for opposite-sex stimuli (A, B) and same-sex stimuli (D, E) for supraliminal and subliminal conditions. Naked bodies are indicated in red and dressed bodies in black. Arrow indicates the N1 component. Mean N1 values for opposite-sex stimuli in the different conditions are illustrated in the histogram below (C). N1 values for same sex stimuli are shown in (D). **p<.01, error bars indicate the standard error.

Within the supraliminal presentation mode, the difference between naked and dressed OSS was highly significant with greater amplitudes for naked bodies (t(18) = 5.33, *p*<.001, Fig. 3A,C). In the SSS condition, naked bodies also produced a greater amplitude than dressed bodies (t(18) = 3.13, *p = *.006, Fig. 3D, F).

The OSS and SSS naked bodies showed no significant difference (t(18) = −1.27, *p*>.1).

Pairwise comparison in the supraliminal condition revealed that naked stimuli produced a greater N1 amplitude than dressed ones both for bodies of the opposite and of the same sex. By contrast no difference was observed for naked bodies when comparing opposite and same sex.

#### Amplitude in subliminal condition

The pairwise comparison revealed a significant difference in N1 amplitudes for naked compared to dressed OSS (t(18) = 2.75, *p* = .01, figure 3B, C). On the other hand, the effect was not significant for SSS (t(18) = 0.36, *p*>.1, Fig. 3E, F).

In the subliminal condition, the N1 enhancement for naked OSS compared to naked SSS only showed a statistical trend towards significance (t(18) = −1.39, *p*>.1).

Our pairwise comparison therefore revealed that, in the subliminal condition, naked bodies of the opposite sex produced a significantly greater N1 component compared to dressed bodies of the opposite sex, while this was not observed for bodies of the same sex. However, the comparison of naked bodies of the opposite sex with those of the same sex failed to reach significance.

### Source Localization of the N1 Component

As the ERP N1 analysis for subliminal presentations revealed an amplitude difference only between naked and dressed OSS, subsequent source localization analysis contrasted dressed and naked conditions in OSS and SSS separately.

### Comparing Naked OSS to Dressed OSS

#### Supraliminal condition

**Figure 4:**
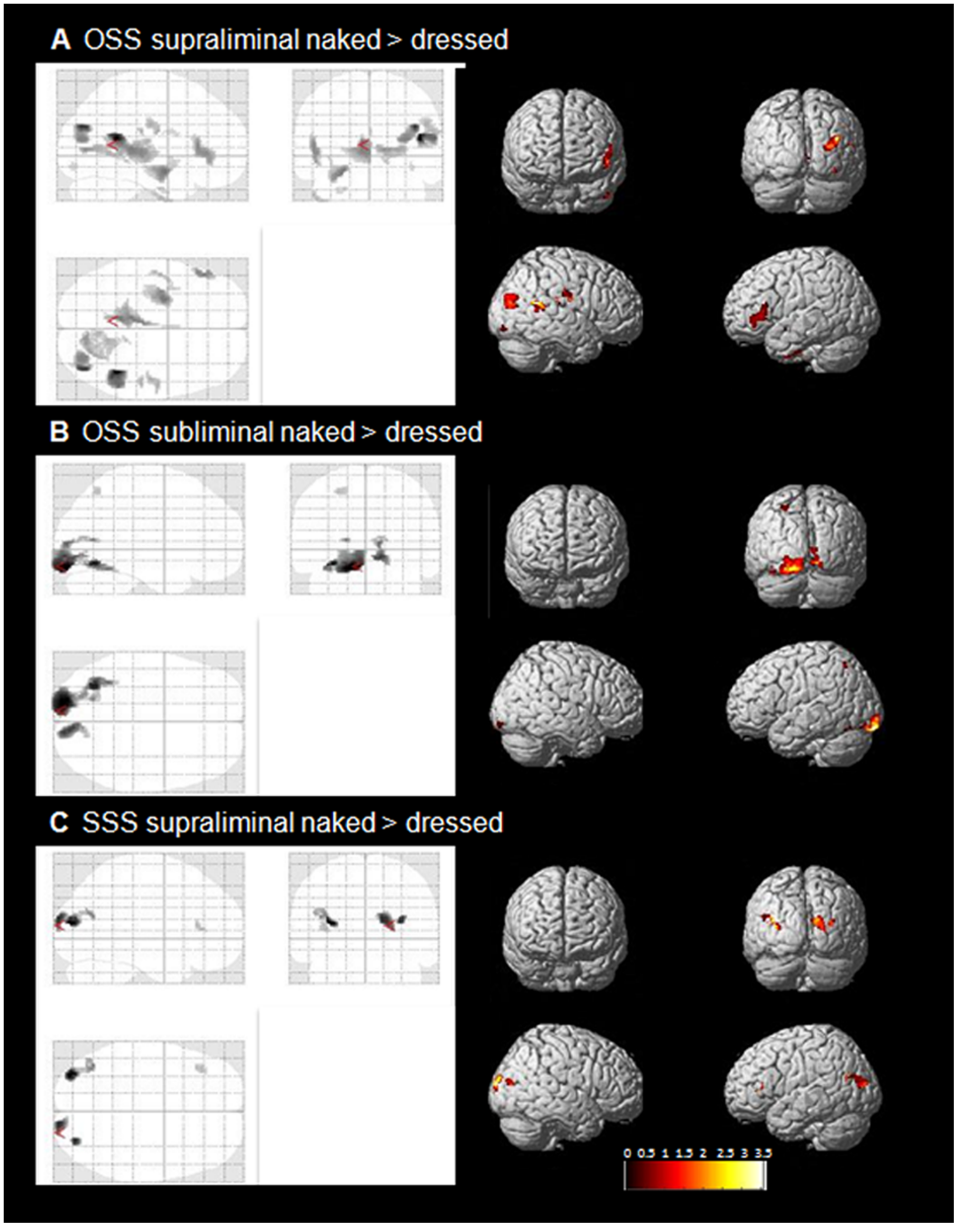
Inverse solution for the N1 component. Inverse solution on the basis of the N1 component estimated using the smooth prior inverse solution implemented in the statistical parametric mapping (SPM8) for the N1 component (146 ms–154 ms). Results show paired t-test for naked compared to dressed opposite/same-sex stimuli in the supraliminal and subliminal condition displayed on a glass brain (xjviewer). Statistical threshold p<0.5, cluster voxel size >15.

Group averaged source estimations of the intracranial generators of the N1 showed that supraliminally presented naked bodies elicited significantly different cortical activation (p<.05) in the right hemisphere. Differences in subcortical activation was confined to the left hemisphere (Fig. 4A, Table 2). The two largest cortical clusters of activation for supraliminal naked OSS were found in the right calcarine sulcus/lingual gyrus (BA17-19) and in the middle occipital right gyrus (BA39). Another cluster of activation in the right hemisphere encompassed the superior and middle temporal right gyri. Additional smaller clusters of activation in the right hemisphere were found in the postcentral and supramarginal gyrus. Important left hemisphere activation was computed in the precuneus, the insula and the inferior frontal gyrus. The cortico-subcortical cluster of activation triggered by the presentation of naked OSS had its maximum peak level activation in the left amygdala, forming a cluster with significant hippocampus and fusiform gyrus (BA20) activation. Additionally, the left thalamus was found to contribute substantially to the processing of naked bodies.

**Table 2:** N1 component inverse solution results.

		peak level						
cluster	cluster size	T	p (uncorr)	MNI (x,y,z)	Label (aal)	Hemisphere		BA
**opposite sex supraliminal naked- dressed**								
1	180	3.56	0.001	44–48 14	Middle temporal gyrus	right		BA21
		2.67	0.008	56–46 16	Superior temporal gyrus			BA22
1	202	2.98	0.004	40–78 26	Middle occipital gyrus		right	BA39
		2.46	0.012	30–80 14				BA19
1	188	2.33	0.016	12–76 4	Calcarine		right	BA18
		2.06	0.027	30–62 6				BA19
		2.2	0.021	24–72 4	Lingual gyrus			BA19
		2.1	0.025	14–56 2				BA18
		2.03	0.028	6–64 8				BA17
		1.92	0.035	26–56 0				BA19
		1.91	0.035	14–40–6				BA30
1	143	2.54	0.01	−26–6–16	Amygdala		left	BA34
		1.88	0.038	−38–16 20	Fusiform gyrus			BA20
		1.86	0.039	−32–22–12	Hippocampus			BA20
1	362	2.51	0.011	−8–44 10	Thalamus		left	BA29
		2.07	0.026	−4–20 12				
		2.43	0.013	−4–26 0				
1	123	2.39	0.014	−50 32 0	Inferior frontal gyrus		left	BA45
		2.38	0.014	−52 26 6				BA45
1	65	2.1	0.025	50–18 28	Supramarginal gyrus		right	BA48
		2.09	0.025	56–10 22	Postcentral gyrus			BA43
1	17	2.06	0.026	54–26 22	Rolandic operculum		right	BA48
1	19	1.89	0.037	−36 4–8	Insula		left	BA48
**opposite sex subliminal naked-dressed**								
1	584	2.46	0.012	−16–92–18	Lingual gyrus		left	BA18
		1.99	0.031	−22–76–8				BA18
		1.86	0.039	−24–82–16	Fusiform gyrus			BA18
1	87	2.34	0.016	−32–64–14	Fusiform gyrus		left	BA19
		2.05	0.028	−36–54–20				BA37
		1.91	0.036	−28–74–14				BA19
		1.82	0.042	−34–46–22				BA37
1	160	2.24	0.019	12–92–8	Lingual gyrus		right	BA18
		2.23	0.02	8–84–10				BA18
		2.1	0.025	10–90 4	Calcarine sulcus		right	BA17
		1.96	0.033	8–76–6	Lingual gyrus		right	BA17
		1.85	0.04	6–78 8	Calcarine sulcus		right	BA17
1	31	1.84	0.041	−26–62 54	Superior parietal lobe		left	BA7
**same sex supraliminal naked-dressed**								
1	91	2.12	0.024	−32–88 14	Middle occipital gyrus		left	BA18
		1.89	0.037	−42–66 20	Middle temporal gyrus		BA39	
		1.82	0.042	−48–70 24				BA39
		1.79	0.044	−38–76 24	Middle occipital gyrus			BA39
1	22	1.8	0.044	−38 32 8	Inferior frontal gyrus		left	BA45
1	121	2.03	0.028	16–98 16	Cuneus		right	BA17
**same sex subliminal naked-dressed**								
–	–	–	–	–	–		–	–

SPM8, N1 component (146 ms–154 ms) inverse solution. Paired t-test results for opposite/same-sex stimuli in the supraliminal and subliminal condition, statistical threshold p<0.5, cluster voxel size >15, aal labeling, Brodmann Area (BA).

#### Subliminal Condition

In the subliminal condition, naked bodies compared to dressed bodies activated the calcarine fissure and the lingual gyrus bilaterally (BA 17 and 18) as well as the left fusiform gyrus (BA19 and 32). Also in the left hemisphere, the superior parietal lobe (BA7) was found to be significantly more activated to naked bodies compared to dressed bodies (Fig. 4B).

### Comparing Naked SSS to Dressed SSS

#### Supraliminal condition

The comparison between same-sex naked bodies and dressed bodies revealed a very weak difference in activation (Fig. 4C). For same-sex stimuli, naked bodies elicited some activation in the middle occipital to middle temporal gyrus (BA 18 and 39) as well as in the right cuneus (BA17).

#### Subliminal Condition

No significant difference in activation between same-sex naked and same-sex dressed bodies was observed.

## Discussion

Our EEG/ERP study confirms that the N1 component produced by human bodies is increased for naked bodies, the latter producing a greater N1 response. In addition, for the first time using an electrophysiological method, this result was also observed under subliminal viewing conditions, with data showing an increased N1 for naked compared to dressed stimuli of the opposite sex from the viewer. Statistical source localization comparing the intracranial generators of the ERPs when viewing opposite-sex naked vs. dressed bodies revealed activation in several primary and associative visual areas including extrastriate and fusiform regions in supraliminal as well as subliminal presentations, with the former producing additional cortical (insula) and subcortical activity (amygdala, hippocampus, and thalamus).

### ERP Results

Previous studies have described a sensitivity of the N1 component to human bodies [36], [38], [39], [40]. For example, Thierry et al. [36] found an N1 response that was present for bodies, body silhouettes and stick figures compared to scrambled stimuli. Elsewhere, this body-sensitive N1 response was shown to be affected by stimulus inversion (as is the case for faces) and was also modulated by the emotional expression conveyed by body posture [38].

Only very recently however, it was also reported that the body-sensitive N1 was enhanced when naked bodies were presented [31], an effect attributed to the higher motivational and affective value of the naked body. Our current study corroborates this finding, confirming the existence of an early modulation of cerebral activity in response to human nakedness, and further demonstrating its presence when stimuli are not consciously reportable. This is in line with the idea that non-conscious processing could be tuned to the biological relevance of the stimuli, as opposite-sex naked bodies constitute a relevant stimulus for heterosexual participants.

As mentioned in the introductory section above, previous behavioral and brain imaging findings have provided evidence that naked bodies are processed in the absence of awareness. The imaging techniques that were used did not allow the timing of activations to be established. Electrophysiological investigations, which possess the necessary temporal resolution to do so, did not specifically aim at studying automatic processes, but instead focused on the effect of sexual stimuli on attention, for example using a rapid serial vision presentation paradigm [41], or involved conscious assessment of the images [29]. Consequently, the temporal effects in these reports were essentially situated in the 200 ms to 600 ms range.

Nevertheless, data from other fields of affective processing lend support to the possibility of an early effect for behaviorally-relevant stimuli. Indeed, previous findings from studies on facial expression of emotion have shown that even when presented subliminally (using backward masking) and thus not consciously detected, fearful expressions produce an ERP enhancement on occipito-temporal electrodes on the N1 component [4], or its anterior, positive counterpart [42]. Although the ERP responses for faces and bodies might not at first glance seem comparable, both stimuli are in fact biologically and socially relevant [43], charged with social and communicative importance [44], [45] and shown to be high attracters of attention [40], [46]. We would therefore conjecture, along similar lines as Hietanen & Nummenmaa [31] that opposite-sex naked bodies (in heterosexual persons) in fact trigger a more powerful response due to their behavioral relevance and their biologically high significance and value as stimuli, consequently giving rise to a strong N1 enhancement. In accordance with findings using emotionally expressive faces, this would extend to include subliminal viewing conditions.

Unexpectedly however, the N1 enhancement in the subliminal condition in the current study was found only for naked bodies compared to dressed bodies of the opposite sex. Indeed, the difference failed to show up as significant in the comparison between same- and opposite-sex naked bodies. One possible cause for the lack of a significant effect may come from our attempts to control the low-level visual features. Our current study used computer-generated greyscale avatars of bodies rather than photographs of natural bodies in order to control for luminance, color, and position, as well as body posture and emotional body expressions. Furthermore, greyscale pictures instead of color images were created to control differences between skin and cloth color. This resulted in rather unnatural stimuli, lacking a certain amount of authenticity, which may therefore have reduced the arousal associated with naked bodies and therefore may have dampened the ERP differences.

### Source Inversion Results

Source localization algorithms computed on the N1 period in the supraliminal condition revealed stronger activations for naked compared to dressed opposite-sex bodies in several structures that play a role in human body processing, visual processing, as well as affective processing.

The inverse solution algorithm revealed increased activation in visual areas including the calcarine area, middle occipital gyrus (BA17) and lingual gyrus, as well as temporal areas. Heightened activation in primary visual areas in addition to the core body processing regions has previously been noted in the field of affective processing [47]. Stimuli depicting erotic acts [15], [16], [17], [18] as well as human bodies expressing an emotion such as fear or happiness were previously reported to lead to greater activation in the inferior occipital gyrus, middle occipital cortex, as well as the fusiform gyrus. Similarly, emotional faces have also been found to produce an increase in these areas [5]. It has been suggested that the heightened activation of visual regions may be the result of recurrent activation from the amygdala. Indeed, an increased response has been found for emotional faces in several visual areas including the extrastriate occipital regions, fusiform gyrus, lingual gyrus and superior temporal sulcus which have been shown to be correlated with amygdala activity [5], [48]. Furthermore, in epileptic patients, amygdala atrophy has been shown to decrease the neuronal enhancement in visual areas suggesting that this heightened visual response cannot occur without an intact amygdala and thus that this structure is necessary to give rise to this effect [7]. Finally, an anatomical link between the amygdala, primary visual and core face processing areas has recently been identified providing structural evidence for these functional correlations [49].

Additional areas revealed by the inverse solution algorithm at this early time period included the insula, ventrolateral OFC and the thalamus. Interestingly, these structures have been reported as belonging to a general sexual interest network. Insular activity has been reported in studies presenting for example erotic film excerpts [16], static sexually arousing pictures [50] and fearful body postures [17]. Mouras et al. [50] reported insula activation to VSS that additionally correlated with concurrent penile plethysmograph response measures. They hypothesized that their bilateral insula activation represented a relay function, assessing the motivational component of the VSS and communicating it to affective processing structures such as the amygdala and the thalamus. Using the same category of stimuli, Redoute et al. [51] reported that hypogonadal patients who present a decreased sexual drive (libido), showed increased regional cerebral blood flow (rCBF) in the right amygdala, insula and left inferior frontal lobe while viewing pictures of sexual intercourse after pharmacological treatment of their endocrine deficiency. The increased insula, amygdala and inferior frontal gyrus rCBF measures were correlated with higher subjective ratings of sexual arousal for these stimuli after treatment. In line with these findings, the role of the insula has been attributed to the mapping of efferent internal motivational signals (visceral responses) and transcribing them to higher cognitive functions such as emotional feelings [21]. Ventrolateral orbitofrontal and thalamic activations have also been associated with sexual arousal, as well as reward processing and were reportedly active in several other studies investigating VSS [52]. Sescousse et al. [53] assessed the overlap and dissociation of functional brain areas in response to reward processing of primarily reinforcing (erotic) stimuli compared to secondary reinforcers such as monetary gains. In this paper, a shape discrimination performed by the participants was rewarded either by an erotic stimulus or a monetary gain. The presentation of the erotic reward activated the posterior lateral OFC (along with the amygdala) whereas the monetary reward activated the anterior lateral OFC. This double dissociation was interpreted from an evolutionary perspective arguing that the phylo- and ontogenetically older, posterior part of the OFC assesses primary reinforcers related to mating choices whereas the newer anterior OFC assesses secondary reinforcers such as monetary gains. A common network, showing no functional divisions between the two reward categories was located in the ventral striatum, midbrain, ACC and the anterior insula. The activation patterns for opposite-sex naked bodies in our study are in line with the idea that primary reinforcements are processed by the posterior lateral OFC (reviewed in [54]), further suggesting that this occurs quite rapidly.

In subliminal viewing conditions, source localization of the N1 component showed significantly more occipital lobe and left fusiform gyrus activation for naked compared to dressed bodies. The enhanced activity in primary visual and body processing areas for naked bodies is in agreement with our hypothesis of an enhanced response for relevant stimuli, however, contrary to our hypothesis, the amygdala showed no response. A previous fMRI study had suggested the presence of amygdala and insula activation for subliminal sexual stimuli [55], however, this observation is not unequivocal. Another report examining subliminal pictures of opposite-sex naked bodies [27] failed to show any amygdala activation for unseen bodies although in this case insular activity was found. This latter investigation also found activation in several other regions including the inferior parietal and frontal lobe, the cingulate gyrus as well as the putamen, however these other areas may reflect activity beyond the N1 as the temporal resolution of this method does not allow such precise temporal moments to be targeted.

The absence of amygdala activation is at odds with our expectations that a rapid nonconscious subcortical pathway could deal with opposite-sex naked bodies due to their behavioural relevance. Rather it would suggest that the amygdala only becomes involved during conscious viewing. Indeed, if subliminal viewing activates regions involved in automatic, bottom-up activation linked to stimulus appraisal, while supraliminal presentations include additional top-down regulatory activation processes as has been suggested elsewhere [27], [56] then one would have to conclude that the amygdala is linked to effortful conscious processing. It remains however that an enhanced electrophysiological response was observed for these stimuli. Investigations from other areas of emotional processing have found similar enhancements of the N1 (e.g. [4], [42]) and evidence has pointed to a role of the amygdala in producing these effects in the electrophysiological domain as well [57]. The presence of an enhanced response thus seems to be dependant on an intact amygdala and would suggest that this structure might be active but to a weaker extent. A weaker activation may have been missed by the source localization algorithm, especially for the estimation of subcortical generators because in view of their deeper location these are more challenging to compute [58].

In summary, body stimuli presented supraliminally elicit a very early N1 component that is enhanced by naked bodies. The source inversion computed on the N1 shows that visual processing of bodies takes place already within 200 ms of presentation. During this time period, the evaluation of the stimuli (OFC, thalamus) and the mapping of the stimuli to internal states (insula) also appear to arise. Most importantly, our data show that naked opposite-sex bodies produce a significantly greater N1 component compared to opposite-sex dressed bodies in the absence of awareness. Thus, our study suggests that the pattern of brain activation previously described for non-conscious emotional faces is also found for sexual stimuli, offering the possibility that naked bodies might also be processed automatically through the rapid subcortical pathway proposed for biologically-relevant stimuli.
